# The Psychedelic Integration Scales: Tools for Measuring Psychedelic Integration Behaviors and Experiences

**DOI:** 10.3389/fpsyg.2022.863247

**Published:** 2022-05-23

**Authors:** Tomas Frymann, Sophie Whitney, David B. Yaden, Joshua Lipson

**Affiliations:** ^1^Psychology and Spirituality Lab, Department of Counseling and Clinical Psychology, Teachers College, Columbia University, New York, NY, United States; ^2^Depth and Clinical Psychology, Pacifica Graduate University, Carpinteria, CA, United States; ^3^Department of Psychiatry and Behavioral Sciences, Center for Psychedelic and Consciousness Research, Johns Hopkins University School of Medicine, Baltimore, MD, United States

**Keywords:** psychedelics, integration, psilocybin, psychedelic integration, harm reduction, psychotherapy, psychedelic, psychedelic therapy

## Abstract

In this study, we describe the development and initial validation of two psychometric scales for measuring psychedelic integration. Psychedelic integration refers to the post-acute period of time following psychedelic drug administration. We created the Integration Engagement Scale (IES) to capture positive behavioral engagement with integration and the Experienced Integration Scale (EIS) to capture internal aspects of feeling integrated. These scales were developed to measure post-acute psychedelic administration dynamics in order to inform the creation of enhanced integration support and to help refine a general conceptual understanding of the construct of psychedelic integration. The scales are brief and face valid instruments designed for practical use in applied and research settings. Scale items were generated and refined using the Iterative Process Model of scale development, with input from psychedelics experts and clinicians. Content validity, internal structure, and reliability were assessed *via* expert surveys, content validity analysis, cognitive interviewing, convergent validity analysis, exploratory factor analysis, and confirmatory factor analysis. The data indicates the scales are valid and reliable measurements of the behavioral and experiential forms of Psychedelic Integration.

## Introduction

Classic psychedelics (e.g., psilocybin and LSD; Nichols, 2016) can occasion effects spanning self-dissolution, the experience of intense emotions, distortion of sensory awareness, and even a sense of death and rebirth (Griffiths et al., 2006, 2008, 2011, 2016, 2018; Johnson et al., 2008; Cook, 2014; Hendricks et al., 2015; Carhart-Harris et al., 2016; Richards, 2016; Yaden and Griffiths, 2020). Such experiences often result in a lasting sense of improved life quality (Griffiths et al., 2006, 2008, 2011, 2016, 2018; Carhart-Harris et al., 2016). However, psychedelic experiences can also be challenging, confusing, and destabilizing, as they can catalyze radical changes, in both immediate states of consciousness and lasting aspects of life (Strassman, 1984; Barrett et al., 2016; Dos Santos et al., 2016). While enhanced wellbeing is a common result of psychedelic use, the psychological changes they induce can range from positive to negative, depending on how they are contextualized and supported (Carbonaro et al., 2016; Gorman et al., 2021). Psychedelic integration, which we define in this article as “The process by which a psychedelic experience translates into positive changes in daily life,” can help to ensure that change resulting from psychedelic experiences happens as beneficially, sustainably, and smoothly as possible, and that risks associated with use are minimized (Tupper et al., 2015).

Psychedelics are currently utilized in a variety of contexts, spanning psychiatric treatment at medical clinics, clinical trials at university research centers, healing ceremonies at retreat centers, guided journeys with underground guides, and recreational use within a diverse range of settings (Rucker et al., 2019; Yaden et al., 2021, 2022). Interest in the field continues to grow exponentially, as research continues to highlight the potential for psychedelics to contribute to lasting improvements in overall wellbeing and high rates of remission for treatment-resistant mental health symptoms (Mahr and Sweigart, 2020). As of 2013, there were 30 million lifetime users of psychedelics in the United States, a number poised to increase with the passing of decriminalization laws and the establishment of medically licensed clinics offering access to psychedelic treatments (Krebs and Johansen, 2013; Pilecki et al., 2021). However, psychedelic integration remains empirically understudied. As stated in the Yale Handbook for Psilocybin-Assisted Therapy, “While psychedelic integration has become a buzzword in psychedelic communities, it remains somewhat vaguely conceived, undertheorized, and, in general, longs for an operational relationship to the problem being treated” (Guss et al., 2020).

The importance of psychedelic integration became apparent at a cultural level in the wake of widespread LSD use in the United States during the 1960s and 70s. Timothy Leary’s slogan, “turn on, tune in, drop out,” captured the zeitgeist of the time—a strong message to split off from society and generally disconnect from social structures, roles, and responsibilities (Riley et al., 2010; Wesson, 2011). This message of encouraged disconnecting was at odds with healthy and adaptive forms of integration. In the aftermath of the festive free love movement, which placed psychedelic culture in opposition to being an engaged member of society, problems related to a reckless approach to life and substance use arose, including young adults falling into homelessness and using addictive drugs, such as methamphetamine, an increase in STD rates, and children being born without resources to be cared for (Wesson, 2011; Belouin and Henningfield, 2018). At the same time, the therapeutic potential of LSD was well documented (Wesson, 2011). Situated within a milieu that did not adequately support the importance of integration, however, cases of misuse and abuse continued and led to the association of psychedelics with risky and irresponsible behaviors (Tanne, 2004; Nichols, 2016). Today, we are in what has been described as a “psychedelic renaissance,” with rates of usage among adults increasing steadily along with the revival of psychedelic research (Krebs and Johansen, 2013; Kelly et al., 2019; Aday et al., 2020; Yockey et al., 2020; Killion et al., 2021; Palamar et al., 2021), warranting caution for both researchers and clinicians (Yaden et al., 2021).

An understanding of the domain of psychedelic integration begins with the meaning of the individual terms, “psychedelic” and “integration.” In the present article, we propose a definition of psychedelic integration that is congruent with the broader meaning of the term “integration,” as it applies to psychedelic experiences. The term “psychedelic” stems from two Ancient Greek words, *psyche* (mind or soul) and *delos* (to reveal, manifest, or make visible)—translating to mind-manifesting (Nichols, 2016). Neurobiologist Daniel Siegel (2016, p. 125) defines integration as “Unifying or connecting previously disconnected parts”. With “integration” as applied to “psychedelics,” the different elements brought into union are the non-ordinary experience occasioned by a psychedelic, on one hand, and the ordinary experience of daily life, on the other.

With the individual terms in mind, for the purpose of this research, we define psychedelic integration as: the process by which a psychedelic experience translates into positive changes in daily life. This definition describes integration as (1) Ongoing (a “*process*” which takes place over time); (2) Connection-Oriented (the “*translation*” of non-ordinary awareness into changes in ordinary life); (3) Helpful (“*positive changes*” identified as the natural output of applied insight); and (4) Embodied (“*daily life*” implying enacted changes in everyday behaviors). Our proposed definition aligns with scientific literature, including the Psychedelic Harm Reduction and Integration model (PHRI; Gorman et al., 2021, p. 8), which states “The goal of integration is to merge the psychedelic experience with the patient’s daily life in a way that helps the patient live a fuller life with less distress”. It also considers aspects of psychedelic integration described in the Yale Manual for Psilocybin Assisted Treatment of Depression, which states that integration is “a means of both making sense and meaning out of the experience, and helping positive changes and insights carry forth into day-to-day life” (Guss et al., 2020, p. 10). Similarly, the proposed definition of integration fits with Gandy et al. (2020, p. 8) description of therapeutic integration sessions as being “intended to support the participant in fully understanding any insights discovered during the session, and applying them to their life going forward”.

## The Present Study

### Overview of the Domain and Subdomains of Psychedelic Integration

**Figure 1:**
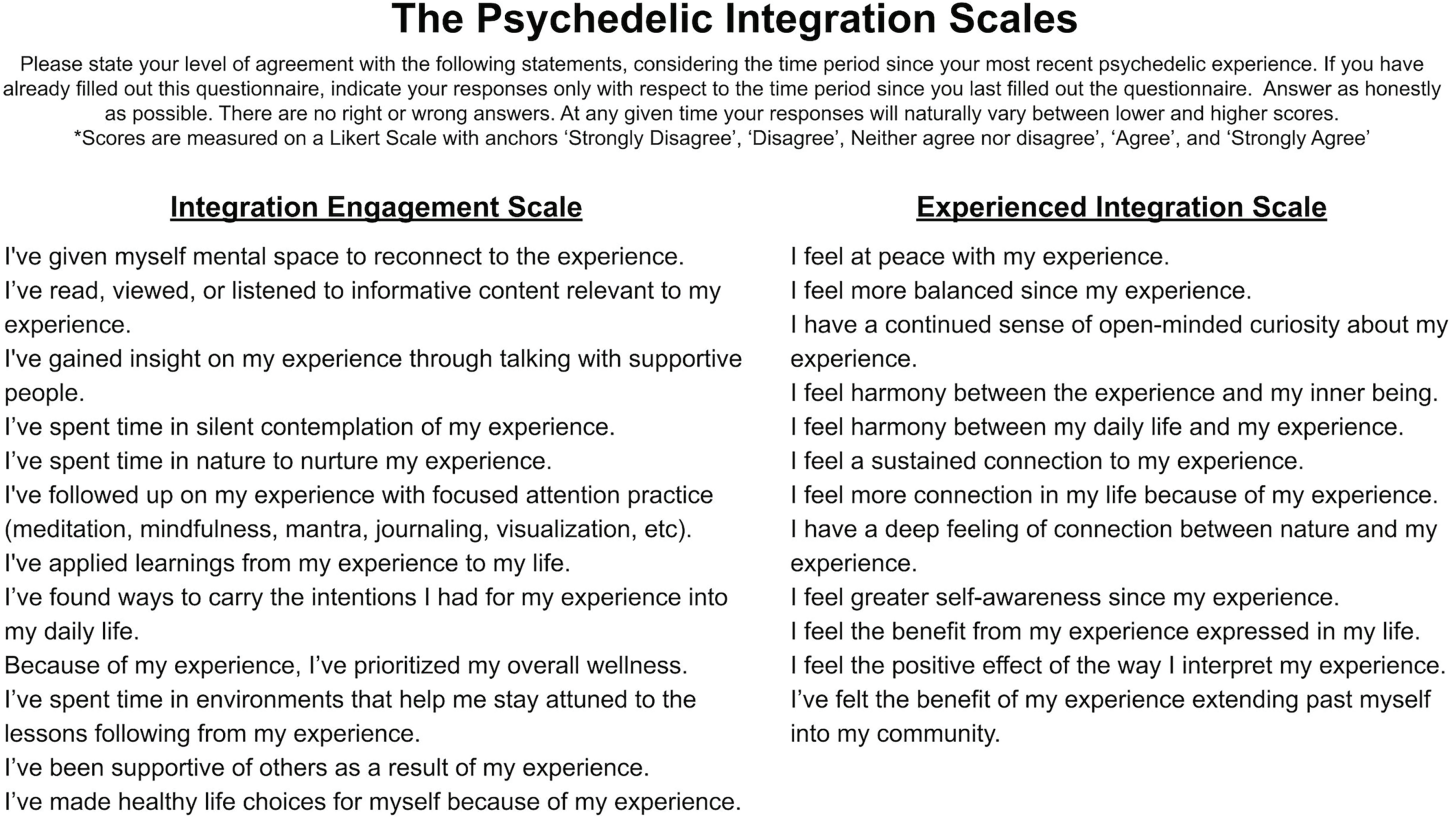
Psychedelic Integration Scales final items.

To measure psychedelic integration, two distinct integration scales were developed, the Integration Engagement Scale (IES) and the Experienced Integration Scale (EIS). The IES focuses on *behavioral* engagement with integration, while the EIS focuses on the *intrapsychic* experience of integration. These scales aim to capture the distinct behavioral and experiential aspects of psychedelic integration, which may or may not co-occur. We developed the scales so that they may either be used individually or in tandem, when contextually appropriate, according to the needs of the investigator. The scales are designed to be used on an ongoing basis, given that psychedelic integration is an ongoing process. The final scale items and instructions are included in Figure 1.

We developed the Psychedelic Integration Scales following the Iterative Process Model, a generalized model of scale development formulated by Chatterji (2003). This approach emphasizes the importance of considering both a theoretical delineation of the measured construct as well as feedback from clinicians and experts, in an iterative process of item revision. The iterative development process of the Psychedelic Integration Scales began with the construction of the Integration Engagement Scale, and after expert feedback subsequently led to the development of a complementary Experienced Integration Scale. Our final aim was to create two brief psychedelic integration scales that would be valid and reliable tools for predicting beneficial outcomes from psychedelic experiences in clinical and non-clinical settings.

**Figure 2:**
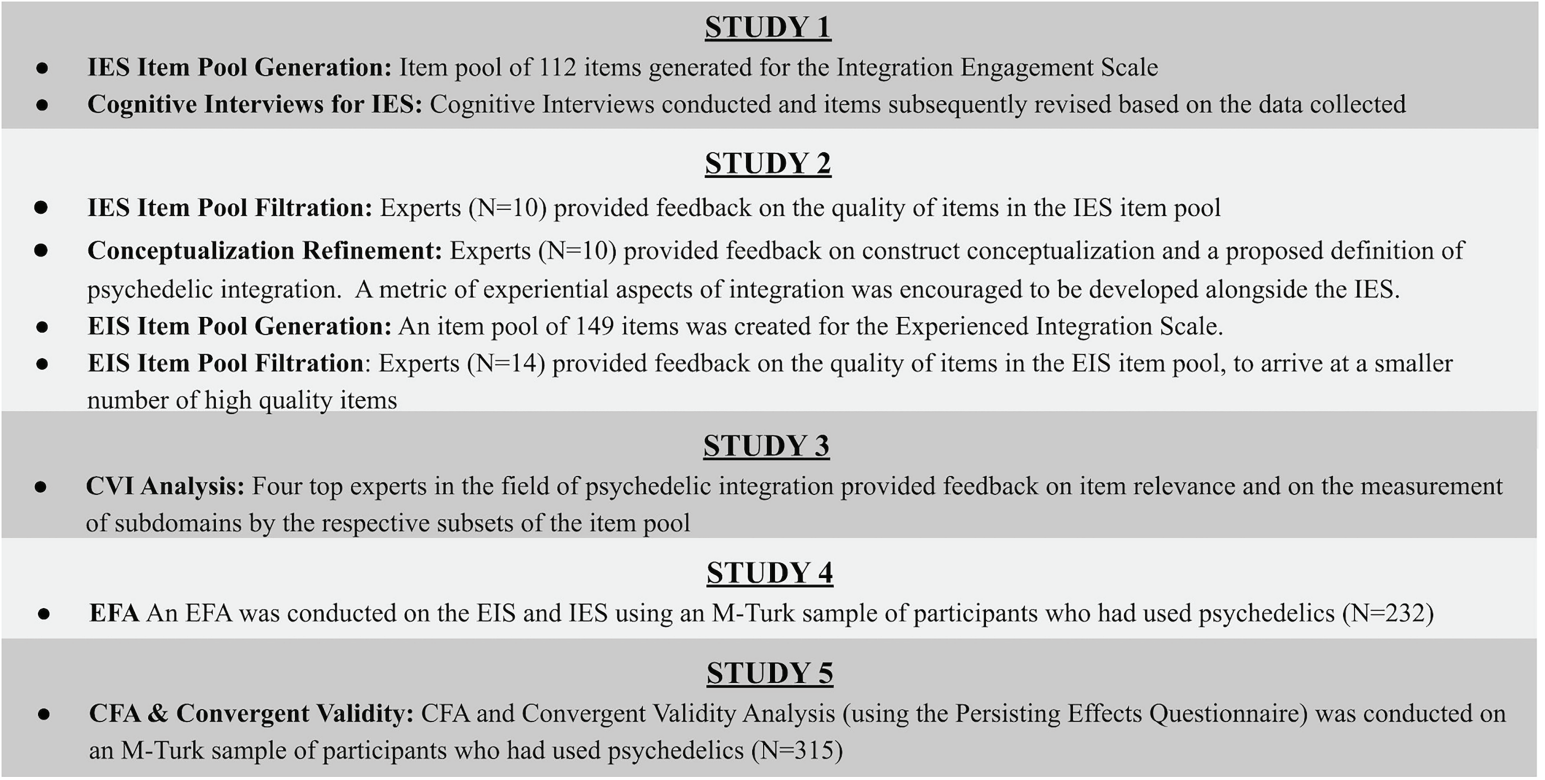
Psychedelic integration scales developmental process outline.

We conducted five studies in order to provide initial validity and reliability evidence on two scales. Pacifica Graduate Institute IRB approved all of these procedures. Figure 2 shows the sequence of developmental steps that were carried out for the Psychedelic Integration Scale.

We delineated the subdomains of psychedelic integration based on a survey of the relevant literature, consultation with experts in the field of psychedelics administration and integration, and a factor analysis. In Study 1, we defined the behavioral domain of “Integration Engagement” as consisting of (A) *Reflection* (contemplative attention given to the experience) and (B) *Application* (behaviors in daily life resulting from awareness gained through the experience). In Study 2, we defined the intrapersonal domain of “Experienced Integration” as feeling (A) *Settled* (at peace with the experience) (B) *Harmonized* (a sense of life alignment with the experience) (C) *Improved* (experiencing tangible benefits). Below is a description of each of the identified subdomains of psychedelic integration.

### Engagement With Integration

#### Reflection

In the aftermath of acute psychedelic experiences, a period of reflection is generally important for making sense of symbolic, emotional, psychological, and spiritual content, and adjusting to potentially radically different ways of understanding the nature of self and reality (Gandy et al., 2020, p. 9; Timmermann et al., 2021). Through reflection, connections are made between aspects of the psychedelic experience and the individual’s life—such as between sudden insights and their life implications, emotional experiences and their psychological underpinnings, or symbolic visions and their personal significance (Gorman et al., 2021). The reflection process may occur through both individual and interpersonal means.

In guided psychedelic experiences the reflection process is usually actively supported. Participants are often encouraged to retrospectively consider and make connections between the intentions that were set pre-session, what unfolded during the session, and how takeaways from the experience could be implemented and sustained in their daily life (Fadiman, 2011; Bourzat and Hunter, 2019; Gorman et al., 2021). Bourzat and Hunter (2019) states, “it is the guide’s role to notice how the intention relates to the content of an experience, reflect these connections, expand the elements that have emerged, and along with the journeyer, track apparent themes,” (p.180). The reflection process is best supported by guides or therapists who are deeply familiar with the effect of the psychedelic used by the participant, who have an empathetic presence, self-awareness, and integrity, and who can support, rather than directly influence, the reflection process (Mithoefer et al., 2016; Phelps, 2017). When the psychedelic experience is held in a group context, reflection may also be supported through structured and informal sharing between group participants.

In addition to reflection supported by guides or therapists, personal reflection can occur through a variety of individual means, such as journaling, silent contemplation, time in nature, reading books, watching videos, or any other means that help further illuminate the experience (Bourzat and Hunter, 2019). While trained guides or therapists may prompt helpful questions and novel perspectives, periods of solitary reflection are inherently suited to the individual attuning their own internal experience and wisdom (Phelps, 2017).

As a whole, the reflection process may be likened to the formation of an internal map for personal growth (Sloshower et al., 2020). In this analogy, the “map” is created through developing self-awareness, which grows and coalesces in the post-experience reflection stage (spanning topics as diverse as childhood experiences, values, personal health, relationships, metaphysics, etc.; Phelps, 2017; Guss et al., 2020). This analogy builds on Payne et al.’s (2021) proposal that psychedelics can, in many cases, serve as a “Compass” on an individual’s mental health journey, initiating, motivating, and steering the course of personal growth. The compass-like function of a psychedelic experience may be primarily related to the experiential content of the journey—the emotions, epiphanies, symbols, etc. that act as “amplifiers of psychotherapeutic practices and processes,” and set the mind in an aligned direction (Gandy et al., 2020, p. 7). While the raw experience may seem to provide a broad compass-like direction for personal growth, the reflection stage is critical to filling in the details for a well-informed path forward on the life journey. In turn, using this analogy, the “application” process can be likened to taking actual steps on the path.

#### Application

“Application,” as used in the present research, refers to putting insights gained from a psychedelic experience into action. Application may be expressed through ongoing daily life choices (such as selection of healthy environments, prioritizing supportive relationships, leaving abusive situations, extending forgiveness, expressing gratitude, and spending time in nature), as well as commitment to a range of different intentional practices (such as meditation, yoga, qi-gong, breathwork, exercise, mindful eating, and prayer; Büssing et al., 2005; Kettner et al., 2019; Guss et al., 2020; Gorman et al., 2021).

Staying connected to the unique intentions set for a psychedelic journey is an important starting point for applying the experience to daily life (Haijen et al., 2018). It may be useful to create intention statements for the psychedelic experience that capture personal values and to engage with values-congruent actions in the aftermath of the experience as a means of integration (Guss et al., 2020). For example, when individuals partake in a psychedelic for the purpose of addiction cessation, translating sobriety-related intentions into a range of supportive actions (such as being selective of keeping company with non-using friends) is an important factor predicting successful recovery (Johnson et al., 2017; Nielson et al., 2018). Overarchingly, the principle of having clear personalized intentions, and connecting those intentions to daily life actions is a broadly relevant and encouraged facet of integration (Sloshower et al., 2020).

In addition to cultivating values by staying connected to intentions and making values-aligned life choices, committed practices or exercises can also support integration and personal growth. Depending on the particular intentions of each individual and the unique nature of their experience, the best-suited practices may vary. For example, loving-kindness meditation may be particularly suited to an individual who has the intention to be more loving, while a practice like dance may be suited to an individual who has the intention to be more embodied and expressive.

Though a variety of practices may be supportive of integration, mindfulness practice has particularly strong empirical support as a beneficial form of psychedelic integration engagement (Sampedro et al., 2017; Griffiths et al., 2018; Walsh and Thiessen, 2018; Smigielski et al., 2019; Heuschkel and Kuypers, 2020; Payne et al., 2021; Radakovic et al., 2021). While Payne et al. (2021) liken psychedelic experiences to a compass for personal growth, they describe mindfulness practice as a vehicle for growth. For the purpose of the current research, we might liken the broad idea of a vehicle for growth to the more general concept of application, with mindfulness practice being a particularly well-built vehicle. Payne et al. identify mindfulness as being particularly effective because of its proficiency in helping to deepen and generalize insights, defuse maladaptive thoughts and behaviors, revitalize values and commitments, and maintain present-mindedness. Generally, each of these aspects of mindfulness practice is helpful in promoting psychedelic integration, regardless of the particular intentions of the individual.

### Experienced Integration

#### Feeling Settled

The dramatic changes in consciousness produced by a psychedelic can be accompanied by the surfacing of repressed psychological content, intensified emotions (fear, confusion, paranoia, awe, gratitude, joy, etc), sudden changes in core beliefs, ego death or ego inflation, unprecedented stillness or invigorated motivation, and other amplified emotional and behavioral changes (Carhart-Harris, 2013; Ortigo and Richards, 2021; Timmermann et al., 2021). The dissipation of emotional extremes and return to internal stability and a feeling of settledness naturally tends to occur as the acute effects of a psychedelic subside, though in some cases imbalances may persist (Carhart-Harris and Friston, 2019). The stabilization of potential emotional imbalances or disturbances is an internal indicator of successful integration.

Intrapsychic imbalances can occur following both extreme negative or positive experiences. On the negative end of the spectrum, Barrett et al. (2016) identified seven dimensions characteristic of so-called “bad trips”: fear, grief, death, insanity, isolation, physical distress, and paranoia. When internal resistance is applied to strong negative emotions the unpleasant state often continues, as captured by the maxim “what you resist persists” (Hayes, 2005; Guss et al., 2020). On the other hand, by constructing a narrative which makes sense of challenging experiences, the accompanying emotions tend to resolve more quickly, and the experience takes on a sense of meaning (Dyck and Elcock, 2020; Gashi et al., 2021).

With euphoric or transcendent experiences, the risk of poor integration may pertain to imbalances in ego-centrism (Anderson et al., 2020). Ortigo and Richards (2021, p. 237) note that “A sense of grandiosity and overconfidence may be a temporary side effect of especially profound and sudden apotheosis,” and caution users about the potential to be “The person who after his first powerful journey, suddenly proclaims themselves to be a messiah or a “shaman”. Achieving settledness in regards to potentially ego-inflating experiences involves accessing a sense of humility (Ortigo and Richards, 2021).

#### Feeling Harmonized

In a broad sense, integration refers to the process of uniting different things. The successful uniting of different states of consciousness is of particular importance in regards to psychedelics use because of the radically non-ordinary states they can occasion, and the consequent change in personal belief structures that may result (Lebedev et al., 2016). The magnitude of change in consciousness is evidenced by brain scan studies of individuals under the influence of psychedelics (Dos Santos et al., 2016; Preller et al., 2019). According to Carhart-Harris’ Relaxed Beliefs Under Psychedelics Model, these changes in brain states tend to increase entropy and decrease the rigidity of beliefs, paving the way for change in belief structures (Carhart-Harris and Friston, 2019).

The beliefs that may flex as a result of psychedelics can be as fundamental as beliefs about the core nature of reality, beliefs upon which many other facets of life rest (Timmermann et al., 2021). Change in such beliefs can initially be jarring—though may also open individuals to unconsidered potentials (Shipley, 2021). The successful formation of novel intrapsychic connections has been postulated to be a core factor underlying positive mental health change following psychedelics use (Siegel, 2009; Carhart-Harris et al., 2018). As a whole, “feeling harmonized” is a sign that an individual has successfully engaged psychological flexibility in the integration process and aligned internal shifts with external behaviors (Sloshower et al., 2020).

#### Feeling Improved

The experience of improvements in wellbeing is inherent to successful integration. Yet psychedelic experiences often entail initial discomfort during the session and integration phase before improvements are felt (Gorman et al., 2021). This may be because of the tendency for psychedelics to elicit the surfacing of repressed content related to the root causes of psychological ill-being (Sloshower, 2018). In general, avoidance, minimization, dismissal, or psychological distancing are common strategies used to temporarily reduce or manage discomfort and are often maladaptive. As delineated in the ACT framework, sustained avoidance eventually contributes to worsening psychological tension, as the root causes of the tension remain unresolved (Kangas and McDonald, 2009; Payne et al., 2021). Such strategies of avoidance are associated with psychological rigidity, which in turn is associated with ill-being (Morris and Mansell, 2018). When psychedelics induce a state in which an individual is unavoidably confronted with the causes of their ill-being, it minimizes the potential for sustained avoidance. If resistance to the repressed content persists, the experience may become increasingly challenging, and the resolution of root causes delayed. On the other hand, adopting the psychological flexibility to confront such content, despite discomfort or unfamiliarity, and contextualize its meaning within one’s life is indicative of integration (Carbonaro et al., 2016; Gashi et al., 2021). The eventual result of such integration is the resolution of the causes previously underlying ill-being and the experience of improved wellbeing.

Psychedelic experiences can also be predominantly enjoyable, with positive feelings pervading most or all of the experience. When it comes to pleasant experiences, successful integration may entail sustaining a connection to that experience, and the qualities underlying the positive emotions (Guss et al., 2020). For example, if universal love is felt strongly during a psychedelic experience, the integration of that experience could involve identifying what was associated with the feeling (perhaps forgiveness, cosmic unity, or inner stillness), and finding ways to connect to those components in daily life.

Notable shifts in personality induced by psychedelics may also lead to various forms of felt life improvement. Findings suggest that psychedelics use can lead to increased openness (MacLean et al., 2011; Erritzoe et al., 2018). Erritzoe et al. (2018) identified two types of openness, both influenced by psychedelic experiences. First, openness to actions, which pertains to not being set in one’s way and willing to try and do new things. Second, openness to values, which involves embodying qualities, such as permissiveness, open-mindedness, and tolerance. Both facets of openness are components of psychological flexibility, which is strongly associated with psychological wellbeing (Wersebe et al., 2018).

The acute post-experience period of improved wellbeing that can follow after a psychedelic experience is often referred to as the “psychedelic afterglow” (Sampedro et al., 2017). The “afterglow” has been described in various ways; “I felt free, carefree, re-energized” (Watts et al., 2017); “The concrete coat had come off” (Watts et al., 2017); “All that day and well into the next, a high pressure system of wellbeing dominated my psychological weather” (Pollan, 2018, p. 254). In some cases, elevated afterglow states can be the result of having addressed and processed difficult unconscious blocks and emotions previously too uncomfortable to approach. The afterglow period has been suggested to offer a window of increased therapeutic potential, in which open and vulnerable reflection is readily available and positive changes can be reified (Murphy-Beiner and Soar, 2020). The occurrence of peak experiences under psychedelics is predictive of long-term positive changes in psychological wellbeing (Roseman et al., 2018). Particular long-term improvements documented include increases in positive mood, prosocial behaviors, empathy, cognitive flexibility, creativity, value alignment, nature-relatedness, spirituality, self-transcendence, and enhanced mindfulness (Griffiths et al., 2006, 2008, 2011, 2016, 2018; Elsey, 2017; Carhart-Harris et al., 2018; Carhart-Harris and Friston, 2019; Kettner et al., 2019; Luoma et al., 2020; Payne et al., 2021). The reification of change through integration practice can transform the afterglow from a fleeting state to a lasting change. Whether through sustaining connection to an already positive state or through growing from facing a challenge, improved wellbeing is a characteristic of openly and mindfully integrating all facets of the psychedelic experience into life (Siegel, 2016, p. 127).

### Study 1

In this study, we identified subdomains and created a list of indicators and sub-indicators for the IES, based on a literature review, preliminary consultations with researchers and clinicians with expertise on psychedelic experiences, and on perusal of personal accounts and case studies of psychedelic integration. We then generated a pool of 112 items for the IES based on the sub-indicators. We subsequently conducted cognitive interviews with a convenience sample of individuals known to have had psychedelic experiences, with the aim of verifying which items were interpreted in a manner congruent with the intent of the question, as well as discovering any potential problems with item wording. At the time of study one, the authors had not yet intended to create a second scale measuring the experiential aspect of psychedelic integration. As such, cognitive interviewing feedback was only gathered for the IES item pool.

**Figure 3:**
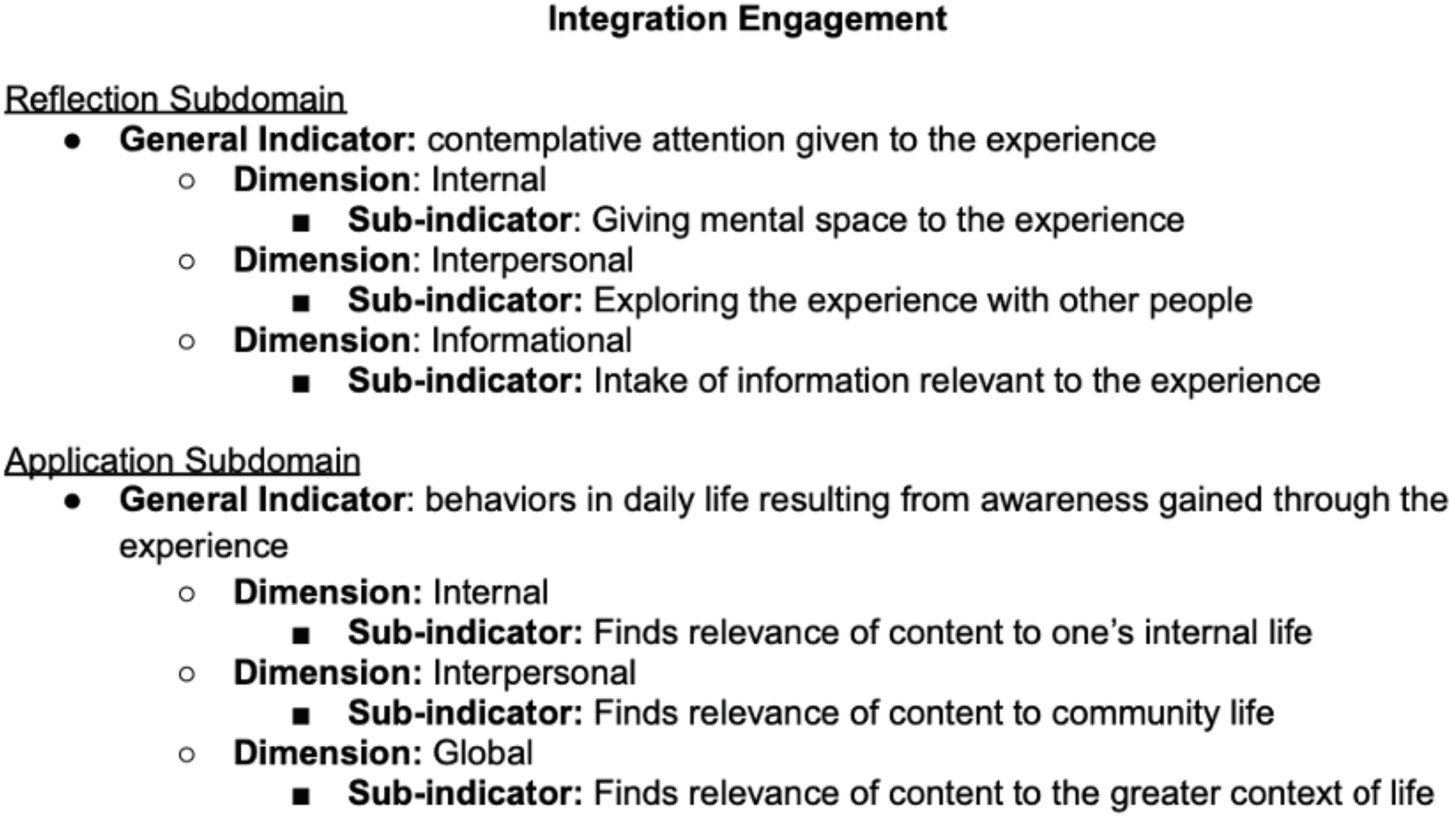
Subdomains, indicators, and sub-indicators for the integration engagement scale (IES).

The following indicators and sub-indicators were used as a basis for the generation of the item pool for the Psychedelic Integration Scales (Figure 3).

#### Participants

A convenience sample of participants known by the authors who’d had psychedelic experiences was recruited either in-person or *via* a phone call (*N* = 13). Participants were adults between the ages of 27 and 58, drawn from the US and were predominantly White (92.3%, *n* = 12; Brazilian 7.7%, *n* = 1), educated (all were high school-educated, and 84.6%, *n* = 11 had bachelor degree or higher), and male (Male = 69.3%, *n* = 9; Female = 30.7%, *N* = 4).

#### Method

Participants were read the scale instructions and told, “Please describe your thought process when responding to the items.” Participants were encouraged to provide more information about relevant aspects of their thought process, using the prompt, “Can you please say more about that?” Responses were transcribed for later analysis.

#### Results

Items were judged and then revised based on the qualitative feedback. 56 revisions were made to the pool of 112 items. Sample changes are listed below (Table 1).

**Table 1:** Item revisions to IES scale items based on cognitive interviewing feedback.

Original item	Identified problem	Revised item
I’ve intentionally given myself space and time to refocus on my experience	The phrasing “given myself space and time” was noted to be ambiguous	I’ve given myself mental space to reconnect to the experience
I’ve read books relating to my experience	Too specific; information intake other than reading may also be helpful	I’ve read, viewed, or listened to informative content relevant to my experience
I’ve communicated about my experience to further my personal growth	The nature of communication was not specified to be helpful to successful integration	I’ve gained insight on my experience through talking with supportive people
I’ve intentionally spent time in nature	The word “intentionally” led many participants to disagree—though they had spent time in nature in a way beneficial to their integration	I’ve spent time in nature to nurture my experience
I’ve taken concrete actions derived from the awareness gained from my experience	The phrase “concrete actions” was overly specific and item was unnecessarily wordy	I’ve applied learnings from my experience to my life
I’ve translated my growth following the experience into supportiveness of others	Unnecessarily verbose wording led participants rereading the question and deliberating on the meaning	I’ve been supportive of others as a result of my experience

#### Discussion

Revisions were made to items that were identified as vague or poetic, to phrasings with interpretations that differed highly between participants, to double-barrelled items, to items that very few individuals agreed with because of a qualifying word, and to items that did not reflect the indicators which they were intended to measure.

### Study 2

**Figure 4:**
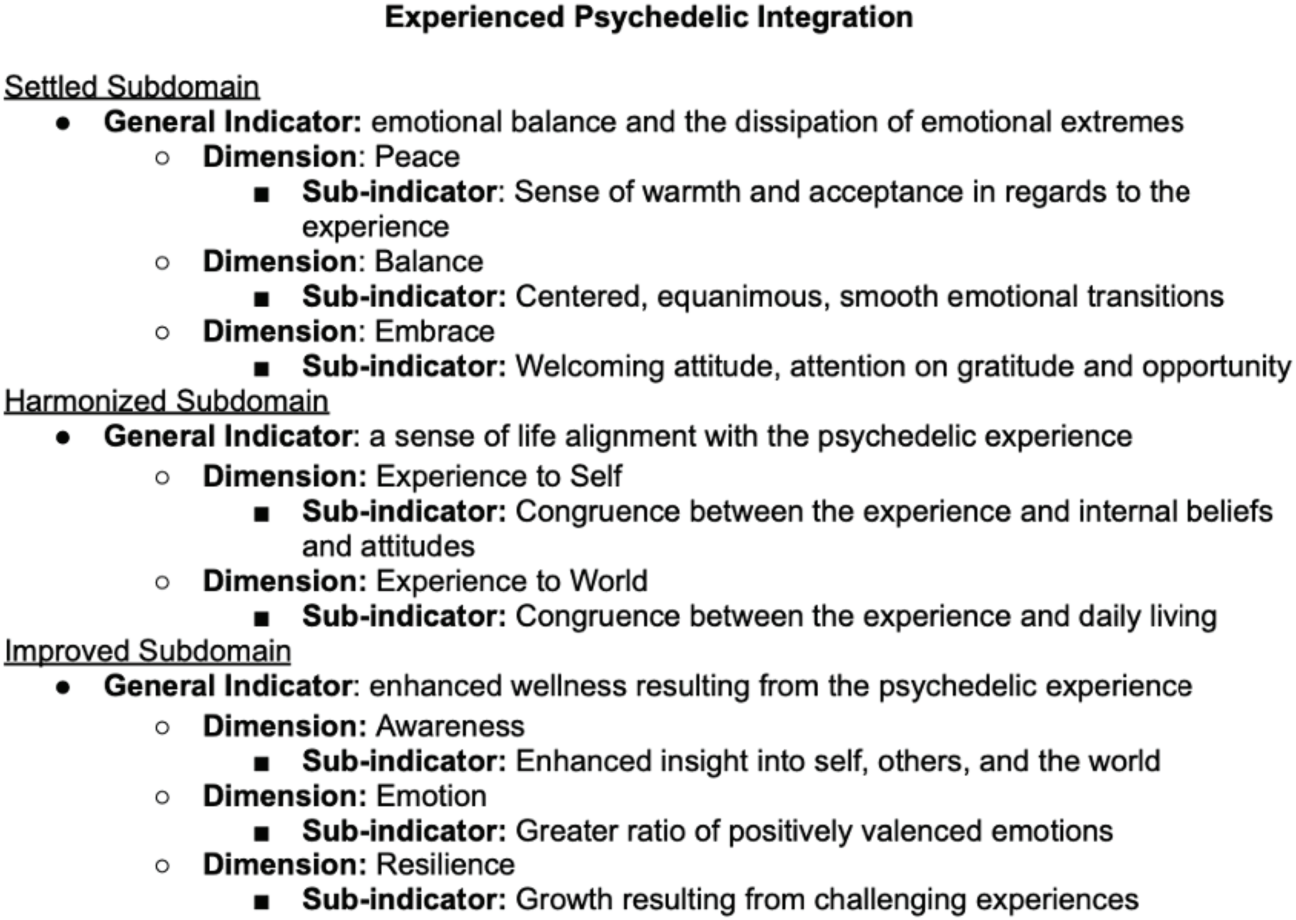
Subdomains, indicators, and sub-indicators for the experience of integration scale (EIS).

In this study, we requested feedback on a proposed definition of psychedelic integration, and administered items to researchers and clinicians with expertise in psychedelic experiences (*N* = 10). Our aim was to improve upon the proposed definition and conceptualization and to arrive at a smaller number of high-quality items to increase construct validity and reduce participant burden. Experts gave feedback on the Integration Engagement Scale items, and also encouraged the construction of a tool to measure the experiential aspects of psychedelic integration. We subsequently identified subdomains and created a list of indicators and sub-indicators for the EIS, based on a literature review, preliminary consultations with researchers and clinicians with expertise on psychedelic experiences, and on perusal of personal accounts and case studies of psychedelic integration. We then created a new item pool of 149 items, which served as the basis of the Experienced Psychedelic Integration Scale (EU = IS). We then reached out to the same set of experts, as well as four additional experts, for feedback on item quality of the newly generated EIS items (*N* = 14; Figure 4).

#### Participants

We recruited researchers and clinicians with expertise on psychedelic integration, reaching out to authors who had published in the field, as well as specialists at psychedelic treatment centers (*N* = 13). Participants were recruited *via* an email message, inviting them to participate in the study.

#### Method

Feedback on definition quality was indicated on a four-point Likert scale, with anchors “Way off,” “Near target,” “On target,” and “Exactly on target,” as well as *via* an open-ended response requesting written feedback on the definition. Feedback on item quality was indicated on a nine-point scale, with anchors “Worthless item,” “Nearly worthless item,” “Very poor item,” “Poor item,” “Mediocre item,” “Good item,” “Great item,” “Excellent item,” and “Perfect item.” Experts were given the instructions “Please indicate whether the following statements is overall a good item to include in the psychedelic integration scale (taking into account the IMPORTANCE, CLARITY, and CONCISENESS of each item). IMPORTANCE: how significant is the aspect of integration captured by this item. CLARITY: how interpretable is this item by a wide range of audiences. CONCISENESS: how succinctly an item is worded.” Items were presented in a single undivided item pool (so that quality could be assessed relative to the overall construct).

#### Results

The definition of psychedelic integration originally proposed was “The intentional application of psychedelic derived awareness into daily life.” Assessment of definition quality indicated an average definition rating of 2.70, falling in between the anchors “Near Target” and “On Target.” Assessment of item quality of the IES item pool indicated an overall average item quality score of 6.80, falling in between the anchors “Good item” and “Great item.” Assessment of item quality of the EIS item pool indicated an overall average item quality score of 7.24, falling in between the anchors “Great item” and “Excellent item.”

#### Discussion

Qualitative feedback regarding the proposed definition of psychedelic integration included encouragements to emphasize: (1) that integration is a process; (2) that intentionality may be a part of the process, but is not a necessary component of all aspects of integration; (3) that the term “application” suggests an overly external view of integration; and (4) that helpful change is inherent to the occurrence of integration. Considerations of the feedback led to the revised definition of psychedelic integration proposed: “The process by which a psychedelic experience translates into positive changes in daily life.”

31 items of the IES item pool had average item quality scores above seven—the anchor corresponding to “Great item.” These items were retained for further analysis. 101 items of the EIS item pool had average item quality scores above seven and were retained for further analysis. Of the 101 items, those that measured the same sub-indicator as another remaining item but had inferior item quality were then eliminated, leaving 31 items in the EIS item pool.

### Study 3

In this study, four top experts in the psychedelic integration field were recruited to provide feedback on the remaining 31 items of the IES item pool and 31 items of the EIS item pool for a content validity index (CVI) assessment. They assessed item relevance, as well as the representativeness of the subsets of the item pool to the measurement of their respective subdomains (Reflection, Application, Settled, Harmonized, and Improved).

#### Participants

Top experts in the field of psychedelic integration were recruited through email requests. Experts consisted of a clinician at Johns Hopkins University, a researcher at New York University, an author who published a book on psychedelic integration, and a psychiatrist at Bristol University.

#### Methods

The relevance of each item to the measurement of psychedelic integration was assessed on a four-point scale, with the anchors “Not relevant,” “Somewhat relevant,” “Quite relevant,” and “Highly relevant.” Item CVI (I-CVI) scores were computed as the number of experts giving the item a 3 or a 4 divided by the total number of experts (Zamanzadeh et al., 2015). The scale CVI (S-CVI) was computed as the overall average of the I-CVI scores for each scale (Zamanzadeh et al., 2015). Representativeness of the collective pool of items measuring each subdomain was measured on a four-point scale with anchors of “Not representative,” “Somewhat representative,” “Quite representative,” “Highly representative.” A subdomain representativeness index was computed as the number of experts giving the item pool a 3 or a 4, divided by the total number of experts. The scale representativeness index was computed as the average of the subdomain representativeness index scores.

#### Results

The final IES demonstrated an S-CVI of 0.96 and a representativeness index of 1.0. The final EIS demonstrated an S-CVI of 0.92 and a representativeness index of 1.0 (Tables 2–5).

**Table 2:** Integration engagement scale CVI results for final scale items.

Integration Engagement Scale (IES)	Item CVI
Reflection	
I’ve given myself mental space to reconnect to the experience	1
I’ve read, viewed, or listened to informative content relevant to my experience	1
I’ve gained insight on my experience through talking with supportive people	1
I’ve spent time in silent contemplation of my experience	1
I’ve spent time in nature to nurture my experience	0.75
I’ve followed up on my experience with focused attention practice (meditation, mindfulness, mantra, journaling, visualization, etc)	0.75
Application	
I’ve applied learnings from my experience to my life	1
I’ve found ways to carry the intentions I had for my experience into my daily life	1
Because of my experience, I’ve prioritized my overall wellness	1
I’ve spent time in environments that help me stay attuned to the lessons following from my experience	1
I’ve been supportive of others as a result of my experience	1
I’ve made healthy life choices for myself because of my experience	1

IES Scale CVI = 0.96.

**Table 3:** Experienced integration scale CVI results.

Experienced Integration Scale (EIS)	Item CVI
Settled	
I feel at peace with my experience	1
I feel more balanced since my experience	1
I have a continued sense of open-minded curiosity about my experience	1
Harmonized	
I feel harmony between the experience and my inner being	1
I feel harmony between my daily life and my experience	1
I feel a sustained connection to my experience	1
I feel more connection in my life because of my experience	1
I have a deep feeling of connection between nature and my experience	0.75
Improved	
I feel greater self-awareness since my experience	1
I feel the benefit from my experience expressed in my life	1
I feel the positive effect of the way I interpret my experience	1
I’ve felt the benefit of my experience extending past myself into my community	1

EIS Scale CVI = 0.98.

**Table 4:** Integration engagement scale representativeness index.

Integration Engagement Scale (IES)	Subdomain Representativeness Index
Reflection	1
Application	1
	Scale Representativeness Index = 1

**Table 5:** Experienced integration scale representativeness index.

Experienced Integration Scale (EIS)	Subdomain Representativeness Index
Settled	1
Harmonized	1
Improved	1
	Scale Representativeness Index = 1

CVI scores for items that were not retained, along with the reason for their elimination, are listed in Study 4, Table 6.

**Table 6:** Item filtration for the integration engagement scale item pool.

CVI	Item kept?	Reason kept/Eliminated	Item
1	Yes	Excellent CVI	I’ve given myself mental space to reconnect to the experience
1	Yes	Excellent CVI	I’ve read, viewed, or listened to informative content relevant to my experience
1	Yes	Excellent CVI	I’ve gained insight on my experience through talking with supportive people
0.75	Yes	Good CVI, important item for capturing nature-based integration, which literature review suggests is important	I’ve spent time in nature to nurture my experience
1	Yes	Excellent CVI	I’ve spent time in silent contemplation of my experience
0.75	Yes	Good CVI, important item for capturing meditation-based integration, which literature review suggests is important	I’ve followed up on my experience with focused attention practice (meditation, mindfulness, mantra, journaling, visualization, etc)
1	Yes	Excellent CVI	I’ve applied learnings from my experience to my life
1	Yes	Excellent CVI	I’ve found ways to carry the intentions I had for my experience into my daily life
1	Yes	Excellent CVI	Because of my experience, I’ve prioritized my overall wellness
1	Yes	Excellent CVI	I’ve spent time in environments that help me stay attuned to the lessons following from my experience
1	Yes	Excellent CVI	I’ve been supportive of others as a result of my experience
1	Yes	Excellent CVI	I’ve made healthy life choices for myself because of my experience
1	No	Redundant: “I’ve spent time in silent contemplation of my experience.”	I’ve spent time in stillness reflecting on the importance of my experience
1	No	Redundant: “I’ve read, viewed, or listened to informative content relevant to my experience.”	I’ve supplemented my experience with helpful written, audio, or visual content
1	No	Redundant: “I’ve spent time in environments that help me stay attuned to the lessons following from my experience.”	I’ve spent time in environments positively aligned with the experience
0.75	No	CVI < 1	I’ve nurtured my experience by spending time in nature
0.75	No	CVI < 1	I’ve read material supportive of personal growth following my experience
0.75	No	CVI < 1	I’ve translated aspects of my experience into positive changes in my daily life
0.75	No	CVI < 1	I’ve shifted away from behaviors that my experience revealed were unhealthy
0.75	No	CVI < 1	I’ve stopped unhealthy behaviors because of my experience
0.75	No	CVI < 1	I’ve been loving toward others as a result of my experience
0.75	No	CVI < 1	I’ve expanded on my experience through spiritually oriented practices
0.75	No	CVI < 1	I’ve removed distractions that take me away from the values I connected to through my experience
0.75	No	CVI < 1	I’ve found ways in daily life to connect to the same qualities that I originally hoped to connect to through the psychedelic
0.75	No	CVI < 1	I’ve engaged in therapy to help work through challenges or further benefits of my experience
0.5	No	CVI < 1	I’ve engaged with mentor or therapist to further the benefit from my experience
0.5	No	CVI < 1	I’ve done daily practices that connect me to awareness accessed during my experience
0.5	No	CVI < 1	I’ve done daily practices to build on my experience
0.5	No	CVI < 1	I’ve done routine practices to build on my experience
0.5	No	CVI < 1	I’ve done physical practices to ground the experience into my body (exercise, yoga, movement, breathwork, dance, hiking, etc.)
0.25	No	CVI < 1	I’ve done physical practices to ground the experience into my body

#### Discussion

Overall, the item and scale CVI scores and the representativeness indexes are excellent. The representativeness index of 1.0 for both scales evidenced that experts were in complete agreement that the set of items capturing each subdomain was highly representative of the subdomain intended to be measured. The content validity index scores indicate that experts were in nearly complete agreement that each of the final items was highly relevant to the measurement of psychedelic integration, with the exception of three items that were rated as “somewhat relevant” by a single expert. Two of those items had to do with nature-based integration, and the third with applied practices. The three lower ratings may have been a reflection of that particular expert’s opinion on the wording of those items, or a lesser priority given to nature-based integration and particular forms of applied integration practice.

### Study 4

In this study, we administered the same 31 items from the IES and 31 items from the EIS item used in the CVI analysis to a sample from the normal population who indicated that they had taken psychedelics. We performed exploratory factor analysis and established initial reliability estimates.

#### Participants

Participants (*N* = 232) were adults (over 18) drawn from the US and were mostly White (79.7%, *n* = 185; Black 11.2%, *n* = 26; Asian 7.7%, *n* = 18), educated (all but one were at least high school-educated; 59.1%, *n* = 137 had bachelor degree or higher), and balanced between females and males (Male = 53.4%, *n* = 124; Female = 46.6%, 108).

#### Method

We collected a sample from M-Turk (an online platform hosted by Amazon Web Services which connects a paid population of respondents with research surveys), using the following language (“Questionnaire for Those Who’ve Had a Psychedelic Experience”). In order to be eligible, our sample of participants needed to have used psychedelics at least once, which was assessed with the question, “Have you ever used a psychedelic substance?” After deleting participants who had not used a psychedelic substance or who failed the attention check (*N* = 129), our final sample was 232. Participants agreed to an informed consent document and confirmed that they were over 18 years of age. Participants were compensated about one dollar for their participation. The survey was administered using Qualtrics, a secure online survey distribution and data collection program.

Participants were asked to consider their most recent psychedelic experience. Specifically, the instructions read: “The following statements have to do with the follow-up to your most recent psychedelic experience. Please indicate the level to which you agree with each statement—considering only the time period since your most recent psychedelic experience. There are no right or wrong answers. Answer as honestly as possible.” Participants were asked specifically to answer regarding the most recent psychedelic experience they had had. Each item was rated on a 7-point scale (1 = Strongly Disagree, 2 = Moderately Disagree, 3 = Somewhat Disagree, 4 = Neutral, 5 = Somewhat Agree, 6 = Moderately Agree, 7 = Strongly Agree).

Participants responded to 31 items from the IES item pool and 31 items from the EIS item pool. The goal was to create a brief scale suited to clinical use. To arrive at a succinct scale with high-quality items, only items with I-CVI scores of 1 were selected from the item pool to be used in the final EFA, with the exceptions of an IES item relating to nature-based integration practice, an IES item relating to focused awareness practices, and an EIS item relating to nature-related integration experience. These items had I-CVI scores of 0.75 but were included in the EFA analysis because they were the only items left in the item pools that captured statements relating to meditation and nature-based integration, both of which were identified in the literature review as particularly important forms of integration engagement. After filtering out items with I-CVI scores below 0.75, items that had CVI scores of 1 but were deemed by the authors to be overly redundant were dropped. The list of the items that were dropped and retained is described in Tables 6 and 7. After item filtration, 12 items remained for both the IES and EIS.

**Table 7:** Item filtration for the experienced integration scale item pool.

CVI	Item Kept?	Reason Kept/Eliminated	Item
1	Yes	Excellent CVI	I feel at peace with my psychedelic experience
1	Yes	Excellent CVI	I feel more balanced since my experience
1	Yes	Excellent CVI	I have a continued sense of open-minded curiosity about my experience
1	Yes	Excellent CVI	I feel harmony between the experience and my inner being
1	Yes	Excellent CVI	I feel harmony between my daily life and my psychedelic experience
1	Yes	Excellent CVI	I feel a sustained connection to my experience
1	Yes	Excellent CVI	I feel more connection in my life because of my experience
0.75	Yes	Good CVI, important item for capturing nature-based integration, which literature review suggests is important	I have a deep feeling of connection between nature and my experience
1	Yes	Excellent CVI	I feel greater self-awareness since my experience
1	Yes	Excellent CVI	I feel the benefit from my experience expressed in my life
1	Yes	Excellent CVI	I feel the positive effect of the way I interpret my experience
1	Yes	Excellent CVI	I’ve felt the benefit of my experience extending past myself into my community
1	No	Redundant with item “I feel the benefit from my experience expressed in my life.”	I feel like my experience has become positively embodied in my life
1	No	Redundant with item “I feel more connection in my life because of my experience.”	I have felt more connected to others since my experience
1	No	Redundant with item “I feel harmony between my daily life and my psychedelic experience.”	I feel a smooth connection between my psychedelic experience and my life
1	No	Redundant with item “I feel greater self-awareness since my experience.”	My experience deepened my awareness of myself
0.75	No	CVI < 1	I feel a warm sense of acceptance toward all aspects of my experience
0.75	No	CVI < 1	My psychedelic experience has made me more connected to a sense of universal love
0.75	No	CVI < 1	My relationships have improved since my experience
0.75	No	CVI < 1	My life surroundings feel as pleasantly aligned with my experience as possible
0.75	No	CVI < 1	My experience feels settled
0.75	No	CVI < 1	I feel disconnected from what my experience showed me
0.5	No	CVI < 1	I wish I had a different experience
0.5	No	CVI < 1	I feel disturbed by my psychedelic experience
0.5	No	CVI < 1	I feel unsettled by my experience
0.5	No	CVI < 1	All the other things in my life have crowded out the impact of my experience
0.5	No	CVI < 1	My life feels out of alignment with my experience
0.5	No	CVI < 1	My experience has faded because I have not put attention on it
0.25	No	CVI < 1	I feel unchanged by my experience
0.25	No	CVI < 1	The way I live feels completely misaligned with my experience
0.25	No	CVI < 1	I feel tension between the experience and my way of life

An EFA was run on these items *via* an M-Turk sample, filtering for only individuals who responded “yes” to the prompting question “Have you ever had a psychedelic experience?” (*N* = 232). Factor solutions were generated using SPSS with a Promax rotation. The oblique promax rotation method was chosen due to the assumption that factors would significantly positively correlate with one another. Parallel Analysis (PA; Horn, 1965), Minimum Average Partialing (MAP; Velicer, 1976), and Scree tests (Cattell, 1966) were used in order to estimate the number of factors, and standards of stability and reliability were applied to drop error factors, and arrive at an adequate factor solution.

#### Results

12 items each remained for both the IES and EIS. Promax rotation of the IES revealed a single-factor model. Scree plots, parallel analysis, and Minimum Average Partialing also pointed to a single-factor model. Promax rotation of the EIS revealed a two-factor model, with the second factor consisting of a single item with a factor loading of above 0.8, “I feel at peace with my experience.” However, Parallel Analysis and Minimum Average Partialing supported a single-factor solution for the EIS. As such, the final Promax rotation was run with the constraint to produce a single-factor solution.

For the IES, a single factor emerged, which accounted for 50.87 percent of the total variance (KMO = 0.932, Bartlett’s sig 0.000). For the EIS, a single factor emerged, which accounted for 56.13% of the total variance (KMO = 0.947, Bartlett’s sig 0.000).

Factor loadings were good (Tables 1 and 2), as all were between 0.6 and 0.8, with the exception of two items from the EIS, which were high (0.817 and 0.814). However, these two items describe two distinct aspects of positive feeling: “I feel the positive effect of the way I interpret my experience” represents the internal expression of positivity, while “I feel the benefit from my experience expressed in my life” represents its external expression (Tables 8 and 9).

**Table 8:** EFA component matrix for the integration engagement scale.

Item	Factor loading
I’ve applied learnings from my experience to my life.	0.794
Because of my experience, I’ve prioritized my overall wellness.	0.783
I’ve found ways to carry the intentions I had for my experience into my daily life.	0.763
I’ve spent time in environments that help me stay attuned to the lessons following from my experience.	0.759
I’ve given myself mental space to reconnect to the experience.	0.731
I’ve spent time in silent contemplation of my experience.	0.725
I’ve made healthy life choices for myself because of my experience.	0.708
I’ve followed up on my experience with focused attention practice (meditation, mindfulness, mantra, visualization, etc).	0.692
I’ve spent time in nature to nurture my experience.	0.661
I’ve read, viewed, or listened to informative content relevant to my experience.	0.644
I’ve gained insight on my experience through talking with supportive people.	0.642
I’ve been supportive of others as a result of my experience.	0.632

**Table 9:** EFA component matrix for the experienced integration scale.

Item	Factor loading
I feel the positive effect of the way I interpret my experience.	0.817
I feel the benefit from my experience expressed in my life.	0.814
I feel more balanced since my experience.	0.794
I feel greater self-awareness since my experience.	0.781
I feel a sense of harmony between my daily life and my psychedelic experience.	0.776
I have a deep feeling of connection between nature and my experience.	0.773
I have a continued sense of open-minded curiosity about my experience.	0.769
I feel more connection in my life because of my experience.	0.749
I feel harmony between the experience and my inner being.	0.712
I’ve felt the benefit of my experience extending past myself into my community.	0.681
I feel a sustained connection to my experience.	0.654
I feel at peace with my psychedelic experience.	0.644

Cronbach’s alpha for the Integration Engagement Scale was 0.90, and Cronbach’s alpha for the Experienced Integration Scale was 0.92.

#### Discussion

Due to an intention to make the scale succinct and practical for use in applied settings, a high constraint was put on redundancy and quality of items. This constraint was likely accountable for the single-factor solution we found in our analysis. Within the single-factor solution of our final scales, the items demonstrated factor scores indicating that each item was pertinent to measurement of the construct, and not overly redundant. Cronbach’s alpha scores demonstrated excellent internal consistency for both scales.

### Study 5

In this study, we administered the IES and the EIS to *N* = 600 participants who reported having had a psychedelic experience in order to assess the convergent and divergent validity of these measures, as well as to conduct confirmatory factor analysis (CFA) to follow up on the results of Study 4’s exploratory factor analysis, which suggested single-factor structures for both the IES and the EIS. We also aimed to assess the convergent validity of the IES and EIS as measures of integration, namely, by measuring their correlation with a 6-item subset of the Persisting Effects Questionnaire (PEQ), an instrument devised by Griffiths et al. (2006) to study the long-term effects of psilocybin. Convergent validity analysis demonstrated strong positive associations with the PEQ item subset, MEQ item subset, and Awe Scale. Divergent validity was supported by a medium correlation with the Satisfaction with Life Scale. Discriminant validity was assessed by comparing the correlation between the EIS and IES and the square root of the AVE. The high correlation between the scales relative to the square root of the AVE did not indicate discriminant validity between the scales.

#### Participants

We collected a sample from M-Turk, under a survey titled Questionnaire for Those Who’ve Had a Psychedelic Experience. Our sample of participants needed to have used psychedelics, which was assessed with the question “Have you ever used a psychedelic substance?” After deleting participants who had not used a psychedelic substance, who failed the attention check, or who wrote incoherent responses to a verbal prompt, our final sample included 315 individuals. Participants agreed to an informed consent document and confirmed that they were over 18 years of age. Participants were compensated $1.25 for their participation. The survey was administered using Qualtrics, a secure online survey distribution and data collection program. The Institutional Review Board at Pacifica Graduate Institute approved this study.

Participants (*N* = 315) were adults (over 18) drawn from the US and were mostly White (80.6%, *n* = 254; Black 12.7%, *n* = 40; Asian 4.4%, *n* = 14), educated (all were at least high school-educated; 70.5%, *n* = 229 had bachelor degree or higher), and broadly balanced between males and females (Male = 58.4%, *n* = 184; Female = 41.6%, *n* = 131). Participants were asked which psychedelic they had most recently used. The majority had most recently used mushrooms (50.2%, *n* = 158), followed by LSD (20.8%, *n* = 66), MDMA (9.0%, *n* = 28), DMT (9.0%, *n* = 28), Ketamine (2.8%, *n* = 9), Ayahuasca (7.0%, *n* = 22), Other (0.6%, *n* = 2), and Iboga (0.4%, *n* = 1). Participants responded to a prompt asking when their most recent psychedelic experience had been, using anchors of 1 = “In the past week,” (23.2%, *n* = 73), 2 = “In the past month” (35.6%, *n* = 112), 3 = “In the past year,” (22.9%, *n* = 72), 4 = “in the past 5 years” (7.6%, *n* = 24), and 5 = “More than 5 years ago” (10.8%, *n* = 34).

#### Methods

To separately test the fit of the 12-item 1-factor models for the IES and the EIS respectively, we used SPSS AMOS structural equation modeling software to compute model chi-square, Comparative Fit Index (CFI), and Root Mean-Square Error of Approximation (RMSEA), all indices of model fit.

Given that a 1-factor model is the simplest CFA model, and serves as the null hypothesis or comparator in factor analyses involving multiple proposed factors, our 1-factor CFAs of the IES and EIS did not require the use of a comparator.

Convergent and divergent validity correlations were assessed between the IES and EIS and items from the PEQ, MEQ, Awe Scale, and Life Satisfaction Scale (Diener et al., 1985; Griffiths et al., 2006; Yaden et al., 2019). To reduce participant burden a subset of items of the PEQ and MEQ were used, rather than the full scales. Six items of the PEQ were selected a priori, each from a different domain of the scale, chosen by the authors as items that would at face value be relevant to the measurement of convergent validity. The six items of the PEQ chosen included “Your appreciation for life has increased,” “You feel more personal integration,” “You now feel more love and openheartedness,” “You have a more positive relationship with others,” “Your behavior has changed in ways you would consider positive since the experience,” and “Spirituality has become a more central part of your life.” Similarly, eight Items of the MEQ were chosen by the authors. These items included “Feeling that you experienced something profoundly sacred and holy,” “Experience of unity with ultimate reality,” “Experience of amazement,” “Feelings of joy,” “Experience of timelessness,” “Being in a realm with no space boundaries,” “Sense that the experience cannot be described adequately in words,” and “Feeling that you could not do justice to your experience by describing it in words.” These items were derived from the MEQ domains of Positive Mood, Sense of Sacredness, Internal Unity, Transcendence of Time and Space, and Ineffability and Paradoxicality. The shortened AWE-S was used, which includes the items “I sensed things momentarily slow down,” “I felt that my sense of self was diminished,” “I had the sense of being connected to everything,” “I felt that I was in the presence of something grand,” “I felt my jaw drop,” and “I felt challenged to mentally process what I was experiencing.”

To assess divergent validity the five-item Satisfaction with Life Scale was used, which includes the items “In most ways my life is close to my ideal,” “The conditions of my life are excellent,” “I am satisfied with life,” “So far I have gotten the important things I want in life,” and “If I could live my life over, I would change almost nothing.”

#### Results

For the IES, CFI was good (0.969) and RMSEA was good (0.032), with 90% confidence intervals of 0 and 0.05. In addition, the chi-square value obtained (71.0) was less than twice the degrees of freedom (54) of the model, further evidence of good fit. Overall, the 1-factor model of the IES demonstrated robust and superior fit.

For the EIS, CFI was good (0.958) and RMSEA was good (0.037), with 90% confidence intervals of 0.014 and 0.054. In addition, the chi-square value obtained (76.7) was less than twice the degrees of freedom (54) of the model, further evidence of good fit. Therefore, the 1-factor model of the EIS demonstrated robust and superior fit.

Moreover, a strong positive association was demonstrated between the PEQ item subset and both the IES (*r* = 0.76, *p* < 0.001) and the EIS (*r* = 0.73, *p* < 0.001), between the MEQ item subset and both the IES (*r* = 0.65, *p* < 0.001) and the EIS (*r* = 0.73, *p < 0*.001), and between the AWE-S and both the IES (*r* = 0.66, *p* < 0.001) and the EIS (*r* = 0.70, *p < 0*.001), indicating robust convergent validity. Divergent validity was demonstrated by correlations between the Satisfaction with Life Scale and the IES (*r* = 0.47, *p* < 0.001) and the EIS (*r* = 0.42, *p < 0*.001).

The correlation between the two scales was 0.83 (*p* < 0.001). The average variance extracted (AVE) for the IES was 0.51, and the square root of the AVE for the IES was 0.71. The AVE for the EIS was 0.56, and the square root of the AVE for the EIS was 0.75 (Tables 10–14).

**Table 10:** Correlation matrix of EIS with related scales.

Scale	EIS correlation	*p*	Discriminant validity: r < sqrt AVE of EIS (0.75)?
IES	0.83	<0.001	No
PEQ (subset)	0.73	<0.001	Yes
MEQ (subset)	0.73	<0.001	Yes
AWE-S	0.7	<0.001	Yes
Satisfaction with Life	0.42	<0.001	Yes

**Table 11:** Correlation matrix of IES with related scales.

Scale	IES correlation	*p*	Discriminant validity: r < sqrt AVE of EIS (0.71)?
EIS	0.83	<0.001	No
PEQ (subset)	0.76	<0.001	No
MEQ (subset)	0.65	<0.001	Yes
AWE-S	0.66	<0.001	Yes
Satisfaction with Life	0.47	<0.001	Yes

**Table 12:** The integration engagement scale.

Integration Engagement Scale (IES)
**Reflection**
I’ve given myself mental space to reconnect to the experience
I’ve read, viewed, or listened to informative content relevant to my experience
I’ve gained insight on my experience through talking with supportive people
I’ve spent time in silent contemplation of my experience
I’ve spent time in nature to nurture my experience
I’ve followed up on my experience with focused attention practice (meditation, mindfulness, mantra, journaling, visualization, etc)
**Application**
I’ve applied learnings from my experience to my life
I’ve found ways to carry the intentions I had for my experience into my daily life
Because of my experience, I’ve prioritized my overall wellness
I’ve spent time in environments that help me stay attuned to the lessons following from my experience
I’ve been supportive of others as a result of my experience
I’ve made healthy life choices for myself because of my experience

**Table 13:** The experience integration scale.

Experienced Integration Scale (EIS)
**Settled**
I feel at peace with my experience
I feel more balanced since my experience
I have a continued sense of open-minded curiosity about my experience
**Harmonized**
I feel harmony between the experience and my inner being
I feel harmony between my daily life and my experience
I feel a sustained connection to my experience
I feel more connection in my life because of my experience
I have a deep feeling of connection between nature and my experience
**Improved**
I feel greater self-awareness since my experience
I feel the benefit from my experience expressed in my life
I feel the positive effect of the way I interpret my experience
I’ve felt the benefit of my experience extending past myself into my community

**Table 14:** Summary of scale statistics.

Scale	Alpha	S-CVI	Scale Representativeness Index	Convergent/Divergent validity correlations
Engagement Scale (IES)	0.90	0.96	1.0	PEQ (6 items) *r* = 0.76, *p* < 0.001 MEQ (8 items) *r* = 0.65, *p* < 0.001 AWE-S (6 items) *r* = 0.66, *p* < 0.001 Satisfaction with Life (5 items) *r* = 0.47, *p* < 0.001
Experience Scale (EIS)	0.92	0.98	1.0	PEQ (6 items) *r* = 0.73, *p* < 0.001 MEQ (8 items) *r* = 0.73, *p* < 0.001 AWE-S (6 items) *r* = 0.70, *p* < 0.001 Satisfaction with Life (5 items) *r* = 0.42, *p* < 0.001

#### Discussion

CFA supported the results of Study 4’s EFA, yielding a single-factor structure for the IES, as well as a single-factor structure for the EIS. Convergent validity analysis demonstrated strong positive associations with PEQ, MEQ, and AWE-S items. Divergent validity was evidenced by moderate associations with the Satisfaction with Life Scale.

The high correlation of 0.83 between the IES and EIS, which was greater than the square root of the AVE for either scale, indicates a lack of discriminant validity between the two scales (Fornell and Larcker, 1981). In other words, the construct of engaging with integration and feeling integrated were highly similar in this sample. The correlation between the two scales may have been particularly high due to the length of time since participant’s most recent use of a psychedelic (on average participant’s most recent use fell just between the anchors of “In the last month” and “In the last year”). Integration engagement is hypothesized to eventually lead to the experience of integration. As such, over time it would be expected that scores on the two scales might converge. In future studies, divergent validity may be supported by an analysis of scores on the two scales at points closer in time to the participant’s recent psychedelic experience. Additionally, the authors hypothesize that divergent validity would be particularly pronounced with first time or novice users of psychedelics (particularly with high dose experiences), for whom the novelty and unfamiliarity of the experience would tend to be greater. In such cases, the experience of integration might be expected to be particularly low soon after the experience (given a stance that integration is often a process that takes time), while engagement would optimally be high (given the stance that engagement would support a novice user in adjusting to the potentially highly unfamiliar experience). Furthermore, discriminant validity may have been impacted by the item selection process. The selection process, which led to a small number of relatively diverse items, may have resulted in relatively lower factor loadings, and in turn a relatively lower AVE. This may have in part accounted for a square root of AVE which was lower than the correlation between the scales.

The Psychedelic Integration Scales items are all worded in a positive direction, with higher scores indicating greater integration. The scales are intended to be helpful tools for facilitators and clinicians to gain a sense of the status of a participant’s integration process. The utility of the scales will be improved as data are collected in clinical trials, indicating participant’s levels of integration and corresponding mental health outcomes. With such data, future research may be able to identify meaningful cutoff scores for levels of integration engagement and experience associated with mental health outcomes. Currently, practical interpretation of integration scales scores is limited to the clinician’s interpretation of scale scores relative to the anchor values. For example, a sum score of 72 on the IES would indicate an average level of “Agree” with each of the statements (suggesting a relatively high degree of integration engagement), while a sum score of 24 on the IES would indicate an average response of “Somewhat Disagree” (suggesting a relatively low degree of integration engagement).

Different facets of integration are expected to be accomplished at different rates, depending on factors, such as the strength of dose administered or the degree of challenging content addressed during the psychedelic experience. As such, a single sum score taken at one time point should be interpreted with caution. Furthermore, integration may be most robustly evidenced when it persists over time. For example, if universal love arose as a core quality of a psychedelic experience, integration would be most robustly indicated if an individual continued to act in accordance with qualities of universal love many months after the session. Given these considerations, we suggest administering the Psychedelic Integration Scales at multiple time points, beginning shortly after the acute stage of the psychedelic experience.

## Overall Discussion

The purpose of our research is to validate a pair of scales measuring psychedelic integration. The definition of psychedelic integration set forth in this article is “The process by which a psychedelic experience translates into positive changes in daily life.” This definition characterizes psychedelic integration as ongoing, helpful, embodied, and oriented toward building connection. The two scales constructed in this article, the Integration Engagement Scale (IES) and Experienced Integration Scale (EIS), respectively measure the *behavioral* and *intrapsychic* aspects of integration. “Reflection” and “Application” are identified as the core features of integration engagement. The internal qualities of feeling “Settled,” “Harmonized,” and “Improved” are identified as the experiential hallmarks of integration.

Given that psychedelic experiences can have a very powerful psychological impact involving non-ordinary states of mind, the integration of these experiences is particularly important. As psychedelics use is on the rise, the construction of these scales is both significant and timely. As of 2022, the first legalization of therapeutic psychedelic use occurred in Oregon, and based on FDA designation of psychedelics as a “breakthrough treatment” legalization is predicted to expand over the coming years (Nutt and Carhart-Harris, 2021). In 2013 there were 30 million lifetime users of psychedelics in the United States alone (Krebs and Johansen, 2013). That number has been increasing at an accelerating rate with a shift in popular culture, current clinical trials, and new research publications that highlight the healing properties of psychedelics and plant medicines (Nutt and Carhart-Harris, 2021).

Given that extensive training is required to be a qualified provider of psychedelics, it is unlikely that the supply of sufficiently good facilitators will keep up with the public demand for psychedelics use. As such, it is reasonable to predict that many unintegrated experiences will result from the hundreds of millions of total psychedelic journeys that will likely occur in the coming years. Within Western and urban cultural containers that have individualistic, consumerist, and performance-oriented values and social structures, the challenges of integrating profound psychedelic experiences may be particularly acute (Diament et al., 2021). It is imperative that the scientific community better understands the processing of these powerful experiences in their aftermath, so that facilitators and users may both maximize the sustainable benefits of psychedelic experiences and minimize irresponsible harm (see Yaden et al., 2021) caused by inducing these experiences without a sufficiently supportive frame.

The psychedelic integration scales have the potential to support both benefit maximization and harm reduction related to psychedelics use. Because addiction to psychedelics is extremely rare, and they are physically safe, the form of harm that may result from their use is primarily psychological—most often related to reactions to the profound changes in consciousness which psychedelics can occasion (Müller and Schumann, 2011; Shalit et al., 2019). In order to improve harm reduction for individuals who choose to have a psychedelic experience, we must better understand how individuals may be supported in embracing the process of change and discovering positive potential within their experiences. “Set and Setting” are proposed to be two fundamental aspects of creating a positive environment conducive to harm reduction and benefit maximization during the experience. We propose that “Reflection and Application” are analogously two fundamental aspects of creating a situation conducive to benefit maximization and harm reduction in the aftermath—with the resulting qualities of feeling “Settled, Harmonized, and Improved” as testaments to successful integration.

The practical utility of the integration scales will grow with data collection. Future research may contribute to the identification of cutoff scores indicating beneficial or concerning levels of post-acute psychedelic integration, an optimal frequency and time scale during which to administer the scales, a sense of the importance of different aspects of integration given the use of different substances, doses, or intentions, and the relevance of the scales to the integration of psychedelic experiences which may have occurred in the past. An initial suggestion for helpful administration of the scales is to administer the EIS within 48 h of the initial experience, followed by administration of the EIS and IES once per week for 8 to 12 weeks. Item answers of “Somewhat Disagree” or below could be flagged as potential areas of concern related to integration. Response to scale items could be used by facilitators or clinicians as a segue into integration therapy or coaching topics.

The framework for understanding and measuring psychedelic integration proposed in this article is intended to help inform the formation of high-quality contexts for the facilitation of psychedelics. Psychedelics are proposed to be treatments which “address the root cause” of mental health issues, and present hopeful alternatives to other forms of medical treatment which may temporarily relieve symptoms but ultimately prove ineffective at causing sustainable positive shifts in mental health (Wheeler and Dyer, 2020). While traditional psychiatric medication often neutralizes symptoms at the expense of grave side effects, psychedelic medicines present opportunities to address symptom causes and pave the way for positive feedback loops of change to occur—with potentially far fewer lasting adverse side effects. However, the actualization of sustainable positive change resulting from psychedelics use depends on the integration of the psychedelic experience into daily life (Payne et al., 2021). For these substances to be used at their full potential—as catalysts of profound improvement—it is necessary that we augment their use with informed integration support.

In current psychedelic research trials, therapy is an integral part of the treatment design. Therapy can be an important part of the integration process; however, psychedelic integration extends beyond just therapy. Important aspects of integration identified include engagement with nature, supportive communities, seasoned psychedelic guides, personal contemplation, and physical and spiritual practices—all of which extend beyond the boundaries of traditional therapy. Each of these important components of psychedelic integration has been present in the ways of life and ceremony seen in indigenous tribes around the world, who have used psychedelics as sacraments for hundreds to thousands of years (Sessa, 2014; Trope et al., 2019; George et al., 2020). It is also important to acknowledge the historical and ongoing occurrence of misappropriation of psychedelic substances and traditions from indigenous communities (George et al., 2019). To continue to ethically research the healing potential of plant medicines we have a responsibility to honor the cultures and sources through which they have arrived in Western culture and to protect the ceremonial and sacred uses of these substances (Sloshower, 2018; Charcuna Institute, 2019).

## Limitations

Different psychedelics have different properties, may be used in different dosages, and evoke different types of experience. Furthermore, each individual’s character, stage of life, and cultural context is unique. As a consequence, the optimal integration of each particular psychedelic experience will vary drastically. In this study, we only assessed the generalized qualities associated with good integration. We also recognize that integration is an unfolding process that takes time.

As such, a sum score derived from the scales should be interpreted with caution. A small dose experience, for example, may demand less intensive engagement with the integration process. An individual who scores low on the engagement scale after taking a small dose should not interpret that result as a testament that they are not practicing good psychedelic integration. Likewise, a large dose may naturally result in temporarily feeling unsettled or out of alignment, as profound changes continue to reverberate in the aftermath of the experience. An individual who initially scores low on items relating to feeling settled or harmonized should not interpret the score as a failure to integrate the experience. Further research will be needed to identify how optimal integration unfolds in the context of unique personalities, conditions, dosages, timeframes, psychedelic substances, and cultural contexts.

There are also limitations pertaining to the data source used in our study. All data was gathered *via* M-Turk users based in the United States, who indicated that they had had a psychedelic experience. M-Turk has been shown to be a credible source of data collection, and an attention check filter was used to increase data quality (Mortensen and Hughes, 2018). However, the pool of participants used is culturally and demographically limited. As such, conclusions regarding the validity of the scales are likewise limited. Furthermore, the CFA indicated that the majority of the M-Turk participants had most recently used classic psychedelics (predominantly mushrooms, LSD, MDMA, DMT, and Ayahuasca). While the authors postulate that the general principles of psychedelic integration would also apply to less commonly used psychedelic substances, the data are most relevant to the use of classic psychedelics. Another limitation pertaining to the data source includes the lack of demographic information regarding experts in studies two and three. Such information would be pertinent to assessing potential biases in the item rating process.

An additional limitation pertains to the theoretical rather than statistically supported nature of the subdomains. The authors prioritized arriving at succinct scale with conceptually diverse items. As such, a strong CVI and redundancy filter was applied to the set of items prior to EFA, potentially contributing to the finding of a single-factor model for both scales (rather than a multifactor model supporting the delineation of the subdomains). The face value assessment of redundancy implemented by the authors may also have introduced bias into the item selection process. Furthermore, the IES and EIS both showed correlations with each other that exceeded the square root of AVE, statistically indicating a lack of discriminant validity (Fornell and Larcker, 1981). This result may reflect a merging over time of integration engagement and experienced integration. Future studies may be employed to discern whether discriminant validity is supported to a greater degree closer in time to a psychedelic experience, or with novice users. The PEQ item subset and the IES also did not evidence discriminant validity, which may have resulted from selection bias in choosing the subset of PEQ items. The *a priori* selection of PEQ and MEQ item subsets based on face value relevance of the items to convergent validity analysis also may have influenced convergent validity correlation results. To gain a more robust metric of convergent validity, the full PEQ and MEQ scales could be used. Lastly, a more robust analysis of convergent validity would be supported by use of the scales in conjunction with the full PEQ, MEQ, and Awe scales.

## Conclusion

We hope that the formation of the integration scales may be one step further in the direction of psychedelics being used in a responsible and beneficial manner. With enhanced value placed on integration, there is greater potential for psychedelic experiences to translate into the beneficial lived actions of psychedelic users, opening the door for a sense of respect and appreciation from others.

## The Psychedelic Integration Scales

A full list of the final IES and EIS scale items, along with instructions, can be found in Figure 1. A five-point Likert scale is used to measure responses, with the anchors “strongly disagree,” “disagree,” “neither agree nor disagree,” “agree,” and “strongly agree.”

The following prompt is used to orient participants to the task:

“Please state your level of agreement with the following statements, considering the time period since your most recent psychedelic experience. If you have already filled out this questionnaire, indicate your responses only with respect to the time period since you last filled out the questionnaire. Answer as honestly as possible. There are no right or wrong answers. At any given time your responses will naturally vary between lower and higher scores.”

## Data Availability Statement

The raw data supporting the conclusions of this article will be made available by the authors, without undue reservation.

## Ethics Statement

The studies involving human participants were reviewed and approved by Pacifica Graduate Institute IRB. The patients/participants provided their written informed consent to participate in this study.

## Author Contributions

TF and SW jointly conceived of the presented idea, conducted the literature review, scale item pool generation, domain and subdomain specifications, cognitive interviewing data collection and analysis, the expert validation recruitment, data collection, and analysis, and content validity index data collection and analysis. TF wrote the majority of the article, and conducted the exploratory factor analysis. SW wrote a minority of the article. DY provided edits to the writing and suggestions for data analysis and experimental design. JL provided edits to the writing and conducted the confirmatory factor analysis and convergent validity analysis. All authors contributed to the article and approved the submitted version.

## Funding

Personal funding sourced by TF and SW.

## Conflict of Interest

The authors declare that the research was conducted in the absence of any commercial or financial relationships that could be construed as a potential conflict of interest.

## Publisher’s Note

All claims expressed in this article are solely those of the authors and do not necessarily represent those of their affiliated organizations, or those of the publisher, the editors and the reviewers. Any product that may be evaluated in this article, or claim that may be made by its manufacturer, is not guaranteed or endorsed by the publisher.
